# Improved Detection and Characterization of Copy Number Variations Among Diverse Pig Breeds by Array CGH

**DOI:** 10.1534/g3.115.018473

**Published:** 2015-04-22

**Authors:** Jiying Wang, Jicai Jiang, Haifei Wang, Huimin Kang, Qin Zhang, Jian-Feng Liu

**Affiliations:** * Key Laboratory of Animal Genetics, Breeding, and Reproduction, Ministry of Agriculture, National Engineering Laboratory for Animal Breeding, College of Animal Science and Technology, China Agricultural University, Beijing 100193, China; † Shandong Provincial Key Laboratory of Animal Disease Control and Breeding, Institute of Animal Science and Veterinary Medicine, Shandong Academy of Agricultural Sciences, Jinan 250100, China

**Keywords:** genomic variation, copy number variations, array CGH, pigs

## Abstract

As a major component of genomic variation, copy number variations (CNVs) are considered as promising markers for some phenotypic and economically important traits in domestic animals. Using a custom-designed 1M array CGH (aCGH), we performed CNV discovery in 12 pig samples from one Asian wild boar population, six Chinese indigenous breeds, and two European commercial breeds. In total, we identified 758 CNV regions (CNVRs), covering 47.43 Mb of the pig genome sequence. Of the total porcine genes, 1295 genes were completely or partially overlapped with the identified CNVRs, which enriched in the terms related to sensory perception of the environment, neurodevelopmental processes, response to external stimuli, and immunity. Further probing the potential functions of these genes, we also found a suite of genes related important traits, which make them a promising resource for exploring the genetic basis of phenotype differences among diverse pig breeds. Compared with previous relevant studies, the current study highlights that different platforms can complement each other, and the combined implementation of different platforms is beneficial to achieve the most comprehensive CNV calls. CNVs detected in diverse populations herein are essentially complementary to the CNV map in the pig genome, which would be helpful for understanding the pig genome variants and investigating the associations between various phenotypes and CNVs.

Copy number variations (CNVs), defined as gains and losses of genomic sequence greater than 50 bp between two individuals of a species (Mills *et al.* 2011) have been considered as a major source of genomic variation. Since the milestone works by Iafrate *et al.* (2004) and Sebat *et al.* (2004), CNVs have attracted extensive attention in genomic studies of human and other species (Tuzun *et al.* 2005; Redon *et al.* 2006; Alkan *et al.* 2009; Sudmant *et al.* 2010).

In domestic animals, a suite of genes with copy number alteration were found contributing to variation of either phenotypic variability or disease susceptibility, such as the *KIT* gene for white coat phenotype in pigs (Giuffra *et al.* 2002), *SOX5* gene for the pea-comb phenotype in chickens (Wright *et al.* 2009), and *STX17* gene for hair greying and susceptibility to melanoma in horses (Rosengren Pielberg *et al.* 2008). Additionally, the study by Seroussi *et al.* (2010) indicated there were close associations between CNVR#456, located on BTA18, and index of total merit and genetic evaluations for protein production, fat production, and herd life in Holstein cattle. These demonstrated that CNVs can be considered as promising markers for some phenotypic and economically important traits or diseases in domestic animals.

Pig is not only one of the most economically important livestock worldwide, but it represents one of the most important research models for various human diseases (Meurens *et al.* 2012). In the past few years, many efforts have been made to detect CNVs in pig genome based on three types of technologies, *i.e.*, array comparative genomic hybridization (aCGH) (Fadista *et al.* 2008; Li *et al.* 2012; Wang *et al.* 2014), SNP genotyping array (Ramayo-Caldas *et al.* 2010; Chen *et al.* 2012; Wang *et al.* 2012; Wang *et al.* 2013), and genome re-sequencing (Rubin *et al.* 2012; Paudel *et al.* 2013; Jiang *et al.* 2014). Among these technologies, because of their accuracy and cost-effectiveness, aCGHs have remained the most frequently used methods for CNVs identification and genotyping of personal CNVs (Pinkel *et al.* 1998; Feuk *et al.* 2006; Carter 2007). Currently, Roche NimbleGen and Agilent Technologies are the major manufacturers of whole-genome aCGH. Because of their different probe distributions, probe numbers, and workflow, it was found that sensitivity, total number, size range, and breakpoint resolution of CNV calls were different among aCGH platforms (Haraksingh *et al.* 2011).

In our previous studies, we have conducted relevant studies using a custom-designed 2.1M aCGH (Wang *et al.* 2014) and read depth (RD) based on genome re-sequencing (Jiang *et al.* 2014) with samples from diverse pig breeds. To enrich the CNV calling and compare the detection performance between different platforms, we performed an alternative aCGH-based CNV identification by a custom-designed 1M aCGH produced by Agilent Technologies. Identified CNVs herein are essentially complementary to the CNV map in the pig genome, which would be helpful for understanding the pig genome variants and investigating the associations between various phenotypes and CNVs.

## Materials and Methods

### Ethics statements

The whole procedure for collection of the ear tissue samples of all animals was performed in strict accordance with the protocol approved by the Institutional Animal Care and Use Committee (IACUC) of China Agricultural University.

### Selection of pig breeds and animals

In the present study, one Duroc individual was used as the reference, whereas the other 12 individuals selected from diverse populations were used as the test samples. These 12 individuals included one Asian wild pig, two pigs from Yorkshire and Landrace as the representatives of European commercial breeds, and nine unrelated individuals selected from six Chinese indigenous breeds (two Tibetan pigs, two Diannan small-ear pigs, two Meishan pigs, one Min pig, one Daweizi pig, and one Rongchang pig). The illustration of the features of six Chinese indigenous breeds is detailed elsewhere (Zhang *et al.* 1986). Genomic DNA for each of 13 individuals was extracted from the ear tissue using Qiagen DNeasy Tissue kit (Qiagen, Germany).

### Array CGH design, hybridization, and CNV calling

The 1M aCGH was designed and produced by Agilent (Agilent Technologies, Santa Clara, CA, USA) based on the newest build of porcine genome (Sscrofa 10.2) (http://www.animalgenome.org/repository/pig/). It covered 18 autosomes and two sex chromosomes containing 965,080 oligonucleotide probes (60 mers) with a median and average intervals of 2191 bp and 2632 bp, respectively. Genomic DNA labeling, hybridization, and array scanning were performed according to the manufacturer’s instructions.

Raw data were first normalized (algorithm: LOWESS) using Feature Extraction software 10.7 (Agilent technologies). Then, Agilent Genomic Workbench Standard Edition 6.5 software (Agilent Technologies) was used to perform CNV interval detection. The QC metrics motif of Workbench 6.5 ensured adequate quality control of the hybridization data. In present study, an array signal, which met the requirements of intensity value >50 and signal-to-noise ratio >25, was included in the analysis. The Aberration Detection Method 2 algorithm (ADM2), with threshold of 6, bin of 10, and a centralization threshold of 6, was used to identify genomic variation given the log2 ratio of fluorescent signals between test and reference DNA samples. Fuzzy zero correction was carried to prevent inclusion of aberrant segments with low average log2 ratios. Additionally, we applied a relatively stringent postanalysis filter—5 probes, 0.5 log ratio—to ignore small, spurious, or low-quality aberrations. Finally, CNV regions (CNVRs) were determined by aggregating overlapping CNVs identified across all samples according to the criteria previously described (Redon *et al.* 2006).

The raw data generated from aCGH and the sequence information of probes involved in the aCGH designed in our study have been deposited into the GenBank GEO database (GSE49299) (http://www.ncbi.nlm.nih.gov/geo/).

### Quantitative real-time PCR confirmation

Quantitative real-time PCR (qPCR) was used to validate 19 CNVRs identified herein. Primers were designed with the Primer3 web tool (http://frodo.wi.mit.edu/primer3/). The glucagon gene (GCG) is highly conserved between species and has been approved to have a single copy in animals (Ballester *et al.* 2004), and one segment of it was chosen as the control region. To keep the same amplification efficiencies between target and control primers, the PCR efficiencies for all primers used in the study were required to be 1.95–2.10. All qPCR were performed using LightCycler 480 SYBR Green I Master on Roche LightCycler 480 instrument following the manufacturer’s guidelines and cycling conditions. For each sample, we performed validation in duplicate to improve the accuracy of PCR. The second derivative maximum algorithm included within the instrument software was used to determine cycle threshold (Ct) values for each region. The copy number for each test region was calculated using the 2^−ΔΔCt^ method (Livak and Schmittgen 2001), which compares the ΔCt (Ct of the target region minus Ct of the control region) value of samples with CNV to the ΔCt of the reference sample.

### Gene content and functional analyses

Pig CNVRs were annotated using NCBI gene information (ftp://ftp.ncbi.nih.gov/genomes/Sus_scrofa/mapview/seq_gene.md.gz; ftp://ftp.ncbi.nlm.nih.gov/gene/DATA/GENE_INFO/Mammalia/Sus_scrofa.gene_info.gz). Gene Ontology (GO) terms and Kyoto Encyclopedia of Genes and Genomes (KEGG) pathway analyses were performed with the DAVID bioinformatics resources 6.7 (http://david.abcc.ncifcrf.gov/). Because the number of annotated genes in the pig genome is limited so far, we first converted the pig Ensembl IDs to orthologous human Ensembl IDs by BioMart (http://www.biomart.org/) ahead of GO and pathway analyses. Statistical significance was assessed by using the *P* value of a modified Fisher’s exact test and Benjamini correction for multiple testing. Additionally, to test whether genes exhibited a different selective constraint among CNV and non-CNV regions, the dN/dS ratio compared with those human species was computed for each gene, and Wilcoxon rank-sum test was used to test the difference of dN/dS ratios between copy numbers’ varied genes and monomorphic ones.

A clustering analysis for all test samples was performed considering the identified CNVR as genetic makers based on the method reported previously (Gazave *et al.* 2011; Tian *et al.* 2013). Specifically, we first built a scoring matrix of the CNVR data for each individual by encoding a value of “0” or “1” according to the absence or presence of any given CNVR. A hierarchical agglomerative clustering was applied on this matrix of individual vectors using the pvclust function from the pvclust R package (Suzuki and Shimodaira 2006). The agglomerative method chosen was unweighted pair-group method with arithmetic mean (UPGMA). Additionally, 10,000 bootstraps were used to assess the robustness of branches.

## Results

### Genome-wide CNVs identified among diverse pig breeds

In total, we identified 2276 CNVs, with 189.67 CNVs per individual. After merging the overlapping CNVs across different samples, a total of 758 CNVRs (Supporting Information, Table S1) were finally determined, covering 47.43 Mb of the pig genome and corresponding to 1.69% of the genome sequence. The length of these CNVRs ranged from 7.02 kb to 2635.29 kb, with a mean or a median of 62.58 kb or 20.93 kb, respectively.

The results in Table 1 show that the numbers of CNVR are uneven distributed across the genome of individuals surveyed. The average of CNVs per individual was 189.67, ranging from 65 (Y2) to 291 (Z2). Additionally, out of the 758 CNVRs identified in the study, 55% of them were identified in only one sample, confirming that segregating CNVs exist among these individuals tested. However, there was also a considerable proportion of CNVR (12.01%) with frequency ≥50%. In particular, 11 CNVRs harboring 46 CNVs were found among all test samples (Table S2). Compared with our previous CNV study using the read depth (RD) method based on sequencing (Jiang *et al.* 2014), these CNVRs with 100% frequency in the test samples were reflected by the inherent copy number alteration in the genome of the reference pig *per se*.

**Table 1:** Pig sample information and CNVs detected in every individual

	Types	Breed	Sample ID	Sex	CNV No.	Total Length (Mb)
Reference	European commercial breed	American Duroc	D4	Female	—	—
Test samples	Asian wild boar population	—	A1	Female	180	13.52
	South China type	Diannan small-ear pig	DN1	Male	144	11.90
			DN5	Female	229	12.64
	North China type	Min pig	M2	Female	153	12.14
	Lower Changjiang River Basin type	Meishan pig	MS7	Female	211	9.92
			MS8	Female	224	9.98
	Southwest type	Rongchang pig	R2	Male	262	10.78
	Central China type	Daweizi pig	W1	Female	118	8.30
	Plateau type	Tibetan pig	Z2	Female	291	14.26
			Z5	Female	260	12.05
	European commercial breed	Landrace	C3	Female	139	9.78
	European commercial breed	Yorkshire	Y2	Female	65	10.23
	Mean				189.67	11.29

In our study, stringent criteria with mean |log2 ratio| > 0.5 and five consecutive probes were used to call high-confidence CNVs according to the previous studies (Liu *et al.* 2010; Li *et al.* 2012). Among 12 test samples, two of them (DN1 and R2) were male and the reference (D4) was female. Based on the two sex-mismatched arrays, we assessed the false-positive rate produced under the criteria using a similar method as reported previously (Fadista *et al.* 2010). Theoretically, all segments on chrY should be gains, and all segments on chrX should be loss in the male individuals. Consistent with this theoretic inference, all segments on chrY were actually detected as gains in our analyses. As for chrX, 17 segments (1.05 Mb) were identified with the log-intensity ratio >0, resulting in a false-positive rate of 0.36% [1,045,770/(144,288,218*2)]. This indicates very few false-positive CNVs produced under the current criteria.

### Pattern and size distribution of CNV regions

**Figure 1:**
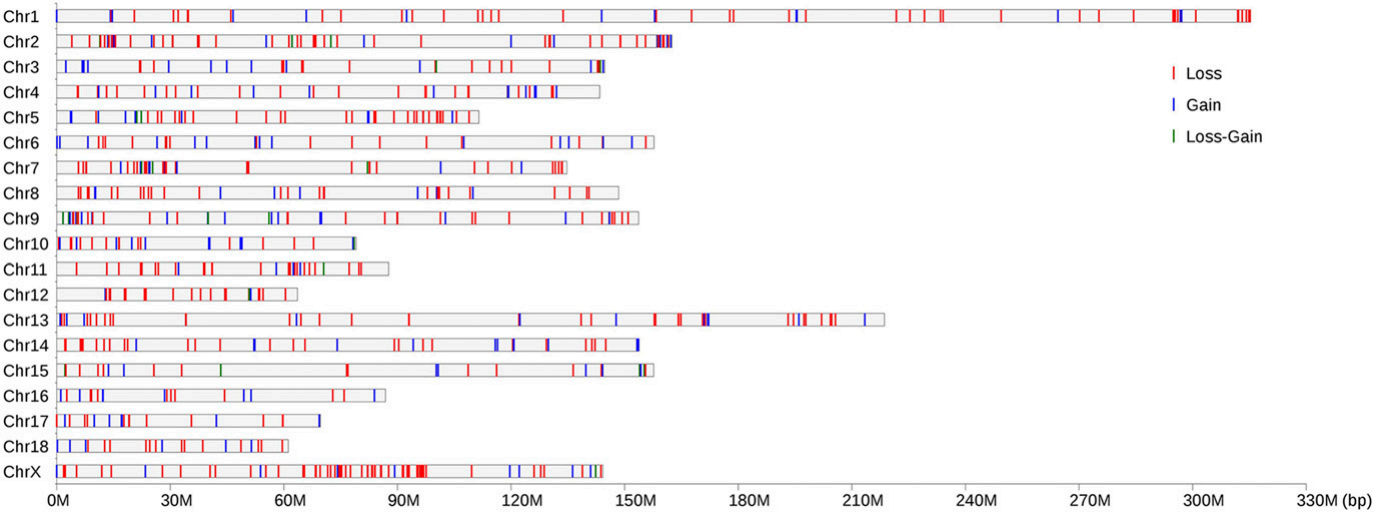
Genomic distribution of CNVRs on 18 chromosomes and chromosome X of pigs. The chromosomal locations of 758 CNVRs are indicated by lines. Y-axis values are chromosome names and X-axis values are chromosome position in Mb, which are proportional to the real size of swine genome sequence assembly (10.2) (http://www.ensembl.org/Sus_scrofa/Info/Index).

**Figure 2:**
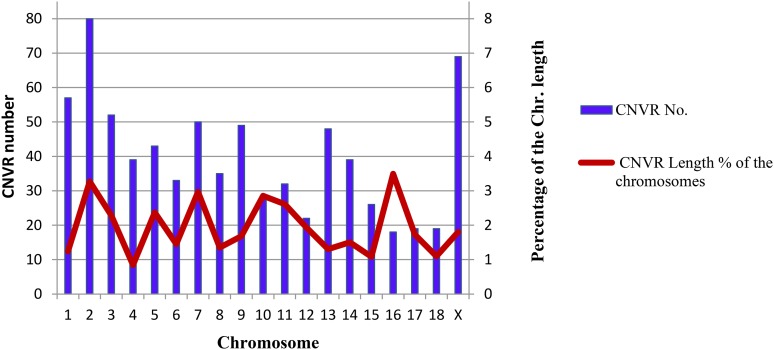
Number and length percentage of CNVRs on the pig chromosomes.

**Figure 3:**
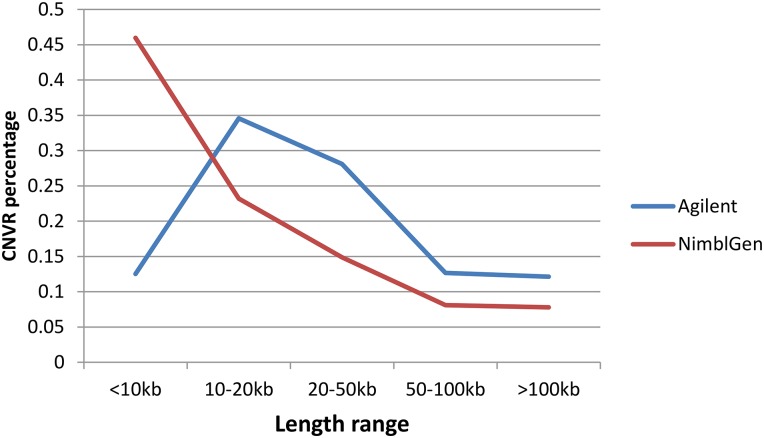
Size distribution of CNVRs identified by aCGH.

Figure 1 illustrates the location and characteristics of all CNVRs identified on the 18 autosomes and chromosome X. Figure 2 further demonstrates that the proportions of CNVR differ greatly across the chromosomes ranging from 0.84% (Chr.4) to 3.49% (Chr.16), with the average of 1.94%. In particular, the proportion of CNVRs in chrX was 1.81%, similar to those in autosomes. Among all 758 CNVRs, the number of loss, gain, and both events (loss and gain within the same region) were 529 (69.79%), 200 (26.39%), and 29 (3.83%), respectively. Loss events were approximately 2.65-fold more common than gain events, but slightly smaller than the size of gain regions on average (45.48 kb *vs.* 80.00 kb). The size distribution of CNVRs (Figure 3) clearly demonstrated that most CNVRs fell into the length interval between 10 kb and 20 kb, and the frequency of CNVRs tends to decrease with the increase of the length.

### Gene content and functional analyses

We sifted out a total number of 1295 porcine genes (Table S3) that were completely or partially overlapped with CNVRs, including 921 protein-coding genes, 343 pseudo genes, 5 miscRNA genes, and 26 genes with other types. These genes overlapped with 355 out of the 758 total identified CNVRs. We further tested the dN/dS ratios for orthologous genes of pig with those of human (Table S4). The results showed that CNVR-related genes have dN/dS ratios significantly higher than those with normal copy numbers by Wilcoxon rank-sum tests (*P* < 6.2E-18).

We further explored if CNVRs identified herein were within duplicated sequence regions or single copy sequence regions with high evolutionary conservation. It is more likely that a CNV of a single copy sequence maintained among other mammals have a phenotypic effect than a CNV at a duplicated sequence. Specifically, we determined those possible single-copy genes in pigs that were also maintained among other mammals based on the resources of one-to-one orthologs in Ensembl (Vilella *et al.* 2009). We sifted out those genes with high orthology confidence and >80% identity in pigs and other mammals, including cow, horse, human, mouse, and sheep. Using these criteria, we finally extracted 1454 of such genes in the pig genome and found that 20 of them were involved in or overlapped with 19 CNVRs (Table S5).

We further performed GO and KEGG pathway analyses for the genes in CNVRs. The GO analyses revealed 53 GO terms (Table S6), of which 12 were statistically significant after Benjamini correction, whereas the KEGG pathway analyses revealed 12 terms (Table S7), of which only one reached statistical significance (Olfactory transduction) after Benjamini correction. The significant GO terms were mainly involved in sensory perception of smell or chemical stimulus, olfactory receptor activity, cognition, G-protein-coupled receptor protein signaling pathway, cell surface receptor–linked signal transduction, antigen processing and presentation, and other basic metabolic processes.

**Figure 4:**
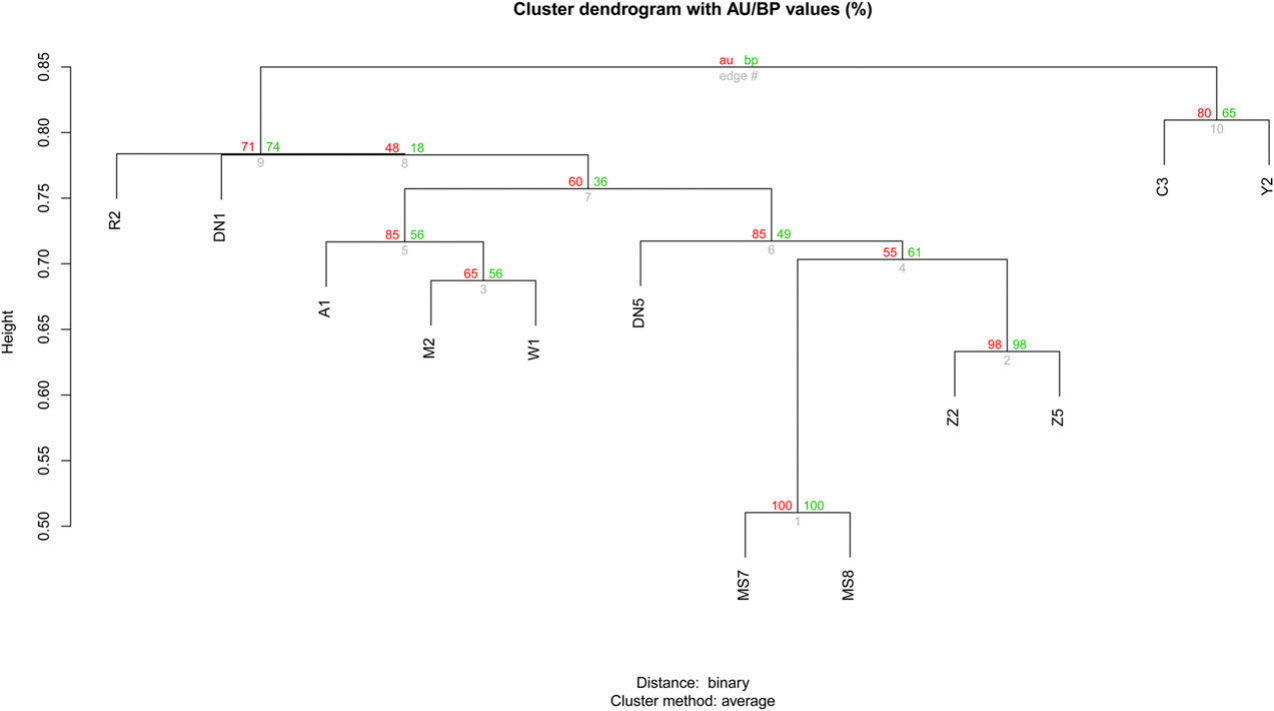
Dendrogram generated by clustering of all the individuals tested on the basis of their CNVR similarities. Numbers at the upper left and upper right of the node and beneath the nodes indicate *P* value, bp values, and edge numbers, respectively.

We also performed the clustering analysis based on the status of CNVRs carried by each test individual. The resulting dendrogram, together with the bootstrap values of every branch (most of them ≥50), is given in Figure 4. The two pigs of European breeds and all other Chinese-originated pigs were clearly divided into two distinct clusters. Within the cluster of Chinese pigs, the genetic relationships of nine individuals tested were basically consistent with that geographical distribution, and two of the three pairs of samples of the same breeds (MS7 and MS8, Z2 and Z5) grouped together. This cluster analysis implies that CNVs may provide information as useful markers in the cluster analysis.

### Quantitative real-time PCR confirmation

From the total 758 CNVRs identified in the study, 19 CNVRs, representing different predicted status of copy numbers (*i.e.*, loss, gain, and both) and different CNVR frequencies (varying from 8.33 to 100%), were chosen to be validated by qPCR. One or two pairs of primers (Table S8) were designed for each CNVR and a total of 31 qPCR assays were performed. Of the 31 qPCR assays, 28 (90.32%) were in agreement with prediction by aCGH. When counting the CNVRs, 17 (89.47%) out of the 19 CNVRs (Table 2) had positive qPCR confirmations by at least one PCR assay. The detailed information of the confirmed 17 CNVRs is listed in Table 2. Our confirmed rate was higher than or similar to those of previous studies (Fadista *et al.* 2008; Ramayo-Caldas *et al.* 2010; Hou *et al.* 2011; Chen *et al.* 2012; Wang *et al.* 2012).

**Table 2:** Results of quantitative real-time PCR analyses of the 17 confirmed CNVRs

CNVR	Chromosome	Start*^a^*	End*^a^*	Type	Frequency	Primer	Positive Samples			Negative Samples		
							Sample No.	Sample Confirmed	Positive Predictive Rate	Sample No.	Samples Confirmed	Negative Predictive Rate
**43**	1	284433601	284517960	Loss	91.67	CGH1-1	11	11	1.0000	1	1	1.0000
						Gene2-2	11	11	1.0000	1	1	1.0000
**47**	1	296124751	296565810	Loss	75	E1-2	9	3	0.3333	2	0	0.0000
**72**	10	22215991	23388810	Loss	58.33	LD1-2	7	7	1.0000	5	3	0.6000
						LD1-3	7	7	1.0000	5	3	0.6000
**105**	11	62691810	62798160	Gain	100	F1-2	12	12	1.0000	0	0	—
						F1-3	12	12	1.0000	0	0	—
**108**	11	62977560	62993310	Loss	16.67	RD2B-4	2	2	1.0000	10	2	0.2000
**114**	11	70562251	71910060	Both	66.67	J1-3	8	8	1.0000	4	1	0.2500
**152**	13	34207351	34233930	Loss	91.67	CGH3-1	11	11	1.0000	1	1	1.0000
						Gene9-4	11	11	1.0000	1	1	1.0000
**339**	2	61350751	61455330	Loss	16.67	R1-3	2	2	1.0000	10	7	0.7000
**418**	3	95980501	95990220	Gain	100	CGH7-1	12	12	1.0000	0	0	—
						CGH7-3	12	12	1.0000	0	0	—
**441**	4	5501251	5544300	Loss	16.67	D1	2	2	1.0000	10	6	0.6000
						D5	2	1	0.5000	10	0	0.0000
**464**	4	99641791	99708810	Gain	58.33	F2	7	6	0.8571	5	2	0.4000
						F4	7	6	0.8571	5	1	0.2000
**579**	7	24621751	24759180	Gain	58.33	RD14A-2	7	7	1.0000	5	3	0.6000
						RD14B-2	7	4	0.5714	5	0	0.0000
**588**	7	50301961	50677050	Loss	16.67	D1-1	2	1	0.5000	11	0	0.0000
						D2-1	2	1	0.5000	11	0	0.0000
**620**	8	43301791	43891650	Gain	16.67	CGH2-1	2	2	1.0000	10	0	0.0000
						kit6	2	2	1.0000	10	0	0.0000
**659**	9	29231131	29275230	Gain	8.33	K5	1	1	1.0000	10	0	0.0000
**734**	X	91336681	91353210	Loss	66.67	CGH6A-2	8	8	1.0000	4	2	0.5000
						CGH5B-1	8	8	1.0000	4	2	0.5000
**736**	X	92583421	92609190	Loss	25	R2-2	3	3	1.0000	8	6	0.7500
Average									0.8971			0.4125

a The Sus scrofa assembly (10.2) (http://www.ensembl.org/Sus_scrofa/Info/Index) was used to indicate the position of the CNVRs.

All 12 test samples and one reference sample in the study were tested in the qPCR assays. Consequentially, we also calculated the positive predictive rates and negative predictive rates for the 17 CNVRs confirmed by qPCR analysis. As showed in Table 2, the average positive predictive rate is 86.54%, demonstrating that, for the positive samples, qPCR assays are highly consistent with the aCGH predictions. Contrary to the positive samples, for some of the negative samples qPCR assays do not agree with the aCGH prediction, *i.e.*, high negative predictive rate (average 42.08%) observed. False-negative identification can be explained by the stringent criteria of CNV calling that minimizes the false-positive but inevitably leads to a high false-negative rate.

### Comparison of CNVRs with those of previous studies

**Figure 5:**
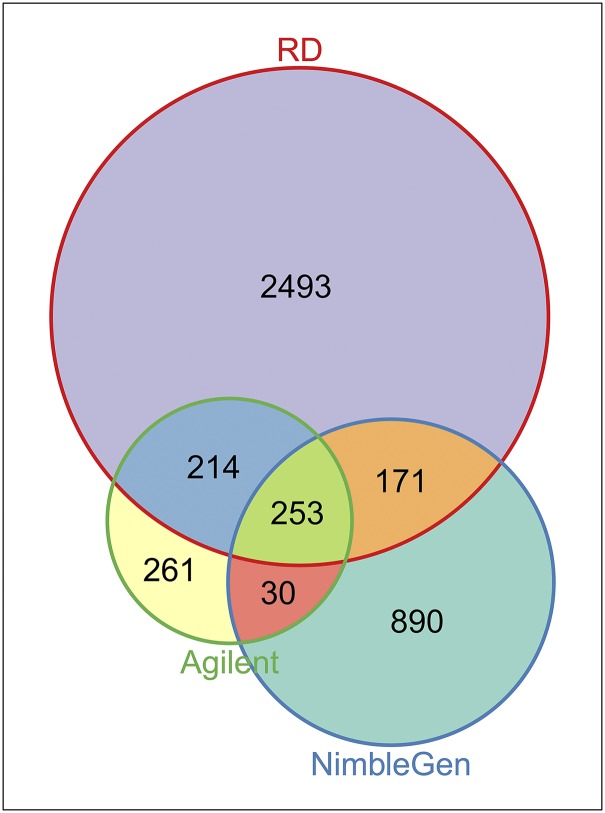
Numbers of CNVs (length) overlapped among different platforms.

CNVRs identified herein were compared with those previously reported in other studies (Fadista *et al.* 2008; Ramayo-Caldas *et al.* 2010; Chen *et al.* 2012; Li *et al.* 2012; Rubin *et al.* 2012; Wang *et al.* 2012; Paudel Y *et al.* 2013; Wang *et al.* 2013; Wang *et al.* 2014; Jiang *et al.* 2014). As shown in Table 3 and Table S9, the CNVR number and length overlapped with the 10 previous studies vary greatly, ranging from 0.13% to 61.61% in count percentage (0.02%–63.06% in length). After merging the comparison results, a total of 526 CNVRs overlap with previously reported ones, indicating 69.39% of CNVRs identified in the study can be validated by previous studies and the other ones were first detected in our study. Furthermore, we performed an exhaustive comparison with our previous studies using 2.1M NimbleGen (Wang *et al.* 2014) and read depth (RD) method (Jiang *et al.* 2014), whereby the same samples were collected in CNV detection. As shown in Figure 5, among the three types of platforms, RD detects the most CNVRs in number or the largest polymorphic sequence in total length, whereas NimbleGen detects more CNVRs in number than Agilent but nearly equal total length. There were 253 CNVRs (length of 17.31 Mb) detected by all three platforms, whereas 261 CNVRs (length of 13.65 Mb) were identified only in the present study.

**Table 3:** Comparison between CNVRs detected in the study with those in the previous reports

Study	CNVR Detected in the Previous Studies							Overlaps in This Study			
	Methods	Sample	CNVRs Detected	Length Range (kb)	Median (kb)	Mean (kb)	Total (Mb)	Count	Count Percentage	Total Length (kb)	Length Percentage
Fadista *et al.* 2008	aCGH (385k)	12	37	1.74–61.92	6.89	9.32	0.43	1	0.13	7.37	0.02
Ramayo-Caldas *et al.* 2010	SNP chip (60k)	55	49	44.65–10,715.82	170.96	754.59	36.97	3	0.40	256.7	0.54
Wang *et al.* 2012	SNP chip (60k)	474	382	5.03–2702.75	142.90	250.69	95.76	10	1.32	678.1	1.43
Li *et al.* 2012	aCGH (720k)	12	259	2.30–1550	98.74	65.07	16.85	55	7.26	1824.2	3.85
Chen *et al.* 2012	SNP chip (60k)	1693	565	50.39–8102.06	252.71	247.55	139.87	106	13.98	10,387.7	21.90
Rubin *et al.* 2012	Genome sequencing	117	1928	0.12–175.50	3.00	5.23	10.08	165	21.77	5217.3	11.00
Wang *et al.* 2013	SNP chip (60k)	14	63	3.20–827.21	97.85	158.37	9.98	14	1.85	2178.3	4.59
Paudel *et al.* 2013	Genome sequencing	16	3118	6.00–96.00	10.00	12.74	39.72	210	27.70	10,996.4	27.18
Wang *et al.* 2014	aCGH (2.1M)	12	1344	3.37–1319.01	11.11	35.56	47.79	283	37.33	21,178.6	44.65
Jiang *et al.* 2014	Genome sequencing	13	3131	10.00–555.13	18.33	32.84	102.8	467	61.61	29,910.8	63.06
This study	aCGH (1M)	12	758	7.02–2635.29	20.93	62.57	47.43	—	—	—	—

Note: The comparison was based on Sus scrofa assembly (10.2). For CNVRs based on the other porcine assembly, we first converted the data to current genome coordinates using the UCSC LiftOver tool (http://genome.ucsc.edu/cgi-bin/hgLiftOver).

## Discussion

Using a custom-designed 1M CGH array, we identified 758 CNVRs among the 12 pig samples from diverse breeds. We found the number of identified CNVR differed among the 12 individuals, and more CNVs per individual were identified in the pigs of Chinese indigenous breeds and wild population (207.2) than in those of modern commercial breeds (100.2). These results are consistent with the previous studies indicating that Chinese indigenous breeds have larger genetic diversity and higher average heterozygosity in comparison with European breeds (Zhang and Plastow 2011), which highlights the importance of using Chinese indigenous breeds in research of porcine genetic architecture.

Concerning copy number status, loss events were approximately 2.65-fold more common than gain events in CNVRs. However, previous studies involving humans have suggested that deletions were more deleterious than duplications, and losses tended to be under stronger purifying selection than gains (Emerson *et al.* 2008; Schrider and Hahn 2010). This observation of more loss than gain events is at least partially related to the technical bias. As also noted by others (Redon *et al.* 2006; Fadista *et al.* 2010), due to the CNV detection pipeline used, the aCGH approach has more power to detect a loss than a gain.

A large amount of annotated genes (1295 genes) are located in the identified CNVRs. CNVR-related genes have dN/dS ratios significantly higher than those with normal copy numbers by Wilcoxon rank-sum tests (*P* < 6.2E-18), which is consistent with the previous results involving pigs and other species (Fadista *et al.* 2010; Li *et al.* 2012). This result indicated that, compared to genes in non-CNV regions, these genes in CNVRs might undertake a different selective constraint and be subjected to a relaxation of constraint due to the redundancy expected from the variable number of gene copies. In accordance with previous studies, GO and KEGG analyses have evidenced that CNVRs are particularly enriched in genes related to sensory perception of the environment (*e.g.*, smell, sight, taste), neurodevelopmental processes, response to external stimuli, and immunity (De Smith *et al.* 2008; Clop *et al.* 2012; Paudel Y *et al.* 2013), suggesting their contribution to adaptation in the wild and behavioral changes during domestication.

Further probing the potential functions of these genes completely or partially overlapped with CNVRs, we also found a suite of promising genes that make them a valuble resource for exploring the genetic basis of phenotype differences among diverse pig breeds. For instance, v-kit Hardy-Zuckerman 4 feline sarcoma viral oncogene homolog (*KIT*) has confirmed that gene duplication and a splice mutation leading the skipping of exon 17 is responsible for the dominant white phenotype (Johansson *et al.* 2005; Pielberg *et al.* 2002). Consistent with the previous studies, in the present study, *KIT* genes were detected to be duplication only in individuals of Landrace and Yorkshire with solid white coat color. Additionally, we sifted 20 single-copy genes, which also maintained one copy among other mammals. These genes with copy number variations are more likely to have a phenotypic effect due to the natural feature of conservation and single-copy status.

The genetic relationships of nine individuals tested using CNVR-based clustering analysis were basically consistent with their geographic distribution, as well as dendrograms generated using microsatellite markers (Fang *et al.* 2005) and high-density SNP data (Jiang *et al.* 2014) in previous studies. Therefore, CNVs may reflect demographic history as Paudel *et al.* (2013) observed in their study, which could be used as genomic markers to investigate pig genetic diversity and evolution. This can be largely reflected by the statistic supports of AU/BP values of resulting branches. It is notable that some branches, *e.g.*, branch 8, obtained values of au/bp less than 50, representing low reliability in the resulting dendrogram. This demonstrates that CNVRs identified in the study are not sufficiently informative in dendrogram construction in contrast to SNPs and other genetic markers. A potential reason lies in that it is not easy to accurately determine the genotype of CNVRs identified by the aCGH platform compared to other genomic variants.

We compared the CNVRs identified in the present study with those in previously studies. As pointed out by Pinto *et al.* (2011), the reproducibility among different platforms varied greatly, and the overlapped rates were affected not only by detecting platforms but also by different samples tested. It is notable herein that both 1M aCGH in the present study and 2.1M aCGH in our previous study (Wang *et al.* 2014) were used for CNV detection with the same samples. These two aCGH have the same coverage on the pig genome but have different densities and positions in probes across the pig genome. The reason we used the same samples in different studies is that we could systematically compare performance of various technique platforms with the same experimental conditions. That comparison of findings by various methods can mostly reflect inherent characteristics of them. Although the density of the 1M aCGH used in the current study is less than that of 2.1M aCGH in our previous study (Wang *et. al*. 2014), the novel 1M probes successfully detected an extra 60 short CNVRs of <10 kb in length (Table S1) that have been previously missed by the 2.1M aCGH as well as RD methods.

Although the present study detected a limited number of novel CNVs compared to our previous studies, the goal of the present study is not only to explore novel CNVs besides our previous findings but also to establish a framework for CNV identification with both high detection power and cost efficiency. Our comparative results between aCGH and RD showed that 1M aCGH by Agilent and 2.1M aCGH by NimbleGen can generate CNV findings complementary to each other, especially in the detection of short CNVs <10 kb. Incorporating findings of present 1M aCGH-based studies and our previous 2.1M aCGH-based studies first proved that ultrahigh-density aCGH can achieve improved performance in CNV calling even more powerful than the sequence-based method in short CNV detection. Our findings herein not only were helpful for constructing a more comprehensive CNV map but also offered a feasible way to design a high-density aCGH with high cost efficiency in CNV studies in contrast to traditional sequence-based methods.

In summary, the present study clearly highlights that different platforms can complement each other, and combined implementation of different platforms is beneficial to achieve the most comprehensive CNV calls. CNVs detected in diverse populations herein are essentially complementary to the CNV map in the pig genome, which would be helpful for understanding the pig genome variants and investigating the associations between various phenotypes and CNVs.

## Supplementary Material

Supporting Information
