# Supplementary material for: Improved Detection and Characterization of Copy Number Variations Among Diverse Pig Breeds by Array CGH
Source: G3 (Bethesda). 2015 Apr 22;5(6):1253–61. doi: 10.1534/g3.115.018473 (PMC4478553; doi:10.1534/g3.115.018473)
Supplement: Supporting Information [file supp_5_6_1253__index.html]

Improved Detection and Characterization of Copy Number Variations Among Diverse Pig Breeds by Array CGH — Supporting Information 

# Improved Detection and Characterization of Copy Number Variations Among Diverse Pig Breeds by Array CGH

## Supporting Information for Wang *et al.*, 2015

**Files in this Data Supplement:**

- Table S1 - Detailed information of each CNVR identified in this study. (.xlsx, 51 KB)
- Table S2 - CNVRs occurred among all test samples with 100% frequency. (.xlsx, 11 KB)
- Table S3 - Annotation of genes in CNVRs detected in this study. (.xlsx, 83 KB)
- Table S4 - dN/dS ratio of genes in CNVRs identified and non-CNVRs. (.xlsx, 1 MB)
- Table S5 - CNVRs affecting single copy genes conserved maintained among other mammals. (.xlsx, 10 KB)
- Table S6 - Gene Ontology of genes in CNVRs identified. (.xlsx, 18 KB)
- Table S7 - Pathway of genes in CNVRs identified. (.xlsx, 10 KB)
- Table S8 - Information of the validated CNVRs and the primers used in quantitative PCR analyses. (.xlsx, 12 KB)
- Table S9 - Detail information between CNVRs detected in the study with those in the previous reports. (.xlsx, 206 KB)
